# Fast and economic immobilization methods described for non-commercial *Pseudomonas* lipases

**DOI:** 10.1186/1472-6750-14-27

**Published:** 2014-04-22

**Authors:** Silvia Cesarini, Belén Infanzón, F I Javier Pastor, Pilar Diaz

**Affiliations:** 1 Department of Microbiology, University of Barcelona, Av. Diagonal 643, 08028 Barcelona, Spain

**Keywords:** *Pseudomonas*, Lipase, Immobilization, Accurel, Celite, FAMEs

## Abstract

**Background:**

There is an increasing interest to seek new enzyme preparations for the development of new products derived from bioprocesses to obtain alternative bio-based materials. In this context, four non-commercial lipases from *Pseudomonas* species were prepared, immobilized on different low-cost supports, and examined for potential biotechnological applications.

**Results:**

To reduce costs of eventual scaling-up, the new lipases were obtained directly from crude cell extracts or from growth culture supernatants, and immobilized by simple adsorption on Accurel EP100, Accurel MP1000 and Celite®545. The enzymes evaluated were LipA and LipC from *Pseudomonas* sp. 42A2, a thermostable mutant of LipC, and LipI.3 from *Pseudomonas* CR611, which were produced in either homologous or heterologous hosts. Best immobilization results were obtained on Accurel EP100 for LipA and on Accurel MP1000 for LipC and its thermostable variant. Lip I.3, requiring a refolding step, was poorly immobilized on all supports tested (best results for Accurel MP1000). To test the behavior of immobilized lipases, they were assayed in triolein transesterification, where the best results were observed for lipases immobilized on Accurel MP1000.

**Conclusions:**

The suggested protocol does not require protein purification and uses crude enzymes immobilized by a fast adsorption technique on low-cost supports, which makes the method suitable for an eventual scaling up aimed at biotechnological applications. Therefore, a fast, simple and economic method for lipase preparation and immobilization has been set up. The low price of the supports tested and the simplicity of the procedure, skipping the tedious and expensive purification steps, will contribute to cost reduction in biotechnological lipase-catalyzed processes.

## Background

According to the Roadmap of the European Commission for Environmental and Sustainable Development (July 2013), “*it is now time to move towards an energy and resource efficient economy, based on the use of nature’s toolbox*”. Bio-inspired processes or enzymatic reactions have a low environmental impact, reduce the amount of waste material and minimize costs, thus serving the requirements to integrate environmental sustainability with economic growth and welfare (Roadmap of the European Commission for Environmental and Sustainable Development, July 2013). Therefore, there is an increasing interest to seek new enzyme preparations and to develop new products derived from bioprocesses to obtain alternative bio-based materials [1,2]. In this context, biotechnological production of biodiesel with lipases has received great consideration in recent years and is undergoing a fast development [3]. Employment of lipases as biocatalysts in the transesterification of triglycerides looks attractive because of the mild reaction conditions required and the easy recovery of glycerol, without an elaborate purification procedure involving production of chemical wastes. Moreover, the enzymatic process tolerates the water content of oil and increases the biodiesel yield by avoiding the typical soap formation caused by alkaline transesterification [4].

Enzyme immobilization is a well reported technology that allows application of enzymes in many biocatalyzed processes like in lipase-mediated biodiesel production [5]. In general, immobilization allows reuse of the biocatalyst, makes the product recovery easier, and frequently enhances the enzyme resistance against inactivation by different denaturants, providing more stable catalysts [6]. Efficiency and recyclability of immobilized enzymes depends not only on the procedure and support utilized but also on the specific enzyme used and the type of process where it is applied [7]. Various immobilization procedures like adsorption, cross-linking, encapsulation or entrapment have been employed on lipases used for biodiesel production [8]. However, most immobilization procedures use sophisticated protocols for lipase entrapment on expensive supports [9], not suitable for a real scale-up, causing an increase in the costs of industrial processes. Therefore, adsorption, used in this work, is a convenient system for lipase immobilization that could be successfully applied to large volume industrial processes, as in biodiesel production, because of its simplicity and low cost [10,11]. This type of immobilization occurs via binding of the lipase onto the surface of the support by weak forces, such as Van der Waals or hydrophobic interactions or through dispersion forces [8]. Adsorbed lipases can be prepared and used under mild conditions without significant activity loss, and the associated process is very simple, as the carrier can be easily recovered for repeated immobilization rounds [5]. Lipases can be adsorbed onto many different supports but generally, porous materials are the best option. These supports allow the effective dispersion of enzyme molecules on a large surface, thus allowing a higher number of enzyme molecules to deliver their catalytic potentiality [12]. In order to obtain stable, efficient and economic enzyme preparations suitable for potential biotechnological applications, in this work three hydrophobic and porous materials were tested as supports for lipase immobilization: two polypropylene matrices (Accurel EP100 and MP1000) and a diatomaceous silica (Celite®545).

Accurel EP100 and MP1000 are commercially available hydrophobic, macroporous, low-density polypropylene powders that display a large surface area for adsorption because of their very small particle size [13,14]. These preparations are extremely resistant towards organic solvents [14-16], a feature that increases their interest for lipase immobilization when aimed at oil transesterification [12,17]. Celite®545 consists of highly porous diatomaceous beads composed of silica (SiO_2_), also containing some other inorganic oxides. Because of its chemical inertness and special interconnected pore structure, Celite®545 constitutes a very suitable support for physical adsorption [18] which has been extensively used in immobilized-lipase biodiesel production [11,19,20].

Previous works aimed at lipase-mediated biodiesel synthesis used mainly commercial enzyme preparations. As an alternative, in the present work we assayed four new lipases from *Pseudomonas* sp. 42A2 and *Pseudomonas* sp. CR-611 [21-23], previously isolated and characterized in our laboratory [24]. Classical enzyme immobilization generally uses purified lipases, a step that requires laboratory materials and equipment that considerably increases the preparation time, thus raising the price of enzyme preparations and the total costs of the process. Therefore, we tried to avoid purification by considering the high hydrophobic nature of lipases and probably of their selective adsorption on the hydrophobic and porous supports used. Accordingly, we describe here the preparation of four non-commercial lipases for potential biotechnological applications. A simple immobilization protocol by fast adsorption of crude enzymes onto three different, inexpensive supports was developed. As a possible application, the behavior of the immobilized lipase preparations was assayed in triolein transesterification for fatty acid methyl esters (FAMEs) production.

## Results

### Lipase production

*Pseudomonas* sp. 42A2 extracellular lipases LipA and LipC were purified and fully characterized in a previous work [21]. LipA is a robust, mesophilic enzyme whereas LipC is more thermolabile and shows a cold-adapted behavior [21,22]. LipCmut, instead, is a LipC thermostable variant previously obtained in our laboratory, still keeping the wild type cold-adapted behavior, thus being a good candidate for biotechnological applications [22,25]. For lipase production, genes coding for LipA, LipC and LipCmut were ligated to pBBR1MCS vector together with their cognate foldase gene *lip*H [21,22]. These constructions were cloned and expressed in the homologous host *Pseudomonas* PABST7.1, where lipases are naturally folded by LipH and further secreted to the culture medium in their fully active conformation [21,22]. Supernatants from each strain were obtained and directly used for activity determination and immobilization. The use of *Pseudomonas aeruginosa* PAO1 mutant strain deficient for chromosomal lipase production (PABST7.1), guarantees that all extracellular lipolytic activity found in supernatants from LipA, LipC or LipCmut recombinant clones corresponds indeed to each individually cloned lipase. Therefore, use of the selected strains as hosts for multicopy plasmid-encoded lipases allowed us to skip the purification steps for each enzyme, thus reducing time and costs of the entire enzyme production process.

*Pseudomonas* sp. CR611 lipase LipI.3 was previously cloned in *E. coli* 5 K, where it is expressed in an inactive form as inclusion bodies [23]. Nevertheless, enzymatic preparations of LipI.3 adequate for evaluation were successfully obtained from inclusion bodies by refolding in urea, as previously described [23]. However, activity recovery from inclusion bodies makes the whole process longer and more expensive, indicating that further improvements of the expression system and enzyme preparation protocol are required to make LipI.3 a faster and more efficient enzyme system. Several attempts to increase Lip I.3 expression were conducted by means of using different vectors and hosts, but in all cases the enzyme was found in the form of inclusion bodies. Consequently, only low but sufficient amounts of active LipI.3 were obtained from the initial construction in order to test this lipase for immobilization on different supports with the same loading charge.

### Effectiveness of lipase immobilization

**Figure 1:**
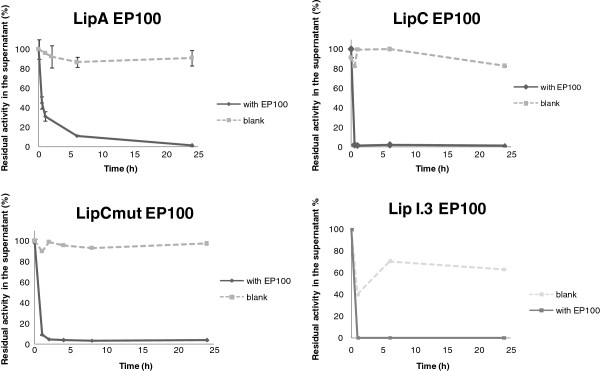
**Immobilization on EP100.** Continuous lines show the decrease of residual activity in immobilization batch supernatants when incubated with the support. As a control, dotted lines show the same supernatants incubated without any support: initial activity was maintained during the 24 h immobilization process, as shown by recovery of initial activity.

**Figure 2:**
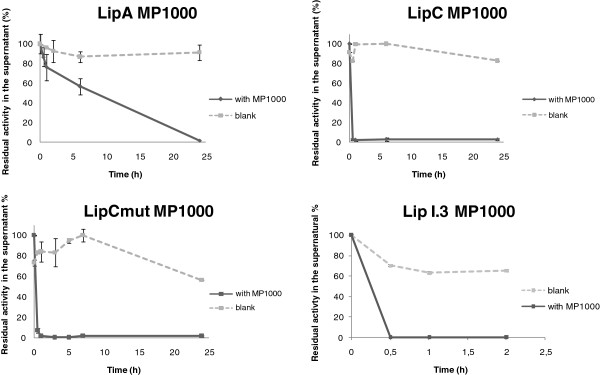
**Immobilization on MP1000.** Continuous lines show the decrease of residual activity in immobilization batch supernatants when incubated with the support. As a control, dotted lines show the same supernatants incubated without any support: initial activity was maintained during the 24 h immobilization process, as shown by almost full recovery of initial activity. Samples were taken mainly during the first 8 h of incubation to better define the rapid decrease of supernatant residual activity. The apparent activity decrease observed for LipCmut control sample was probably due to a different degree of substrate emulsion in samples assayed after 8 h incubation. High immobilization rates were obtained after 2 h incubation.

Adsorption of lipases on hydrophobic polymeric supports has been reported previously [12,13]. However, previous works mainly assayed existing commercial lipases. In this work we wished to discover the behavior of four non-commercial lipases towards immobilization using different kinds of supports, for further biotechnological applications. Thus, direct supernatant samples of PABST7.1 bearing LipA, LipC or LipCmut constructions, and refolded crude cell extracts of *E. coli* LipI.3 were successfully immobilized on EP100, MP1000 and Celite®545. During adsorption on EP100 and MP1000, residual activity corresponding to unbound lipases was measured in supernatants of the immobilization batches as an indicator of the process yield. For this purpose, supernatant samples were obtained at different times, and the decrease of residual activity was plotted for immobilization assessment. As shown in Figure 1 (EP100) and Figure 2 (MP1000), all four lipases tested were efficiently adsorbed on the Accurel matrices used. A fast decrease of residual activity could be observed during the first 5 h incubation for all tested enzymes. For LipC and LipCmut, 90% immobilization was produced within the first hour of incubation, both on EP100 and MP100. Adsorption of LipA on polypropylene matrices proceeded slightly slower, but all lipolytic activity of the batch supernatant was lost after 24 h incubation, indicating a complete immobilization. Enzyme-loaded EP100 and MP1000 were recovered through vacuum filtration and lipase activity of the supports was measured. As shown in Table 1, in general, immobilization on MP1000 resulted in the most active enzyme preparations. Since immobilization on Celite®545 followed a different protocol, only the final activity adsorbed to the silica could be measured (Table 1). Celite®545 showed to be the best support for LipA, although this enzyme also displayed good activity when adsorbed to MP1000. LipC displayed the highest activity values after immobilization on EP100 and MP1000, whereas the catalytic potential of LipCmut and LipI.3 was better preserved on MP1000. Taking into consideration the great variation of activity values found among replica assays (Table 1), and trying to describe a common support for the four lipases tested, it could be concluded that Accurel MP1000 would be the support of choice for immobilization of LipC, LipCmut and LipI.3, whereas LipA would be better immobilized on Celite®545. In general, immobilized lipases displayed full activity over a long period, indicating a good stability of the biocatalysts [10].

**Table 1:** Enzyme activity after immobilization on EP100, MP1000 and Celite®545

	**LipA**	**LipC**	**LipCmut**	**LipI.3**
EP100	6.8 ± 0.8	42.7 ± 1.4	28.6 ± 0.1	34.3 ± 1.1
(U g^-1^ support)				
MP1000	36.0 ± 1.7	39.4 ± 7.8	62.6 ± 0.1	86.2 ± 6.2
(U g^-1^ support)				
Celite®545	48.3 ± 1.0	7.2 ± 3.0	21.7 ± 0.2	13.8 ± 1.9
(U g^-1^ support)				

### FAMEs production

**Figure 3:**
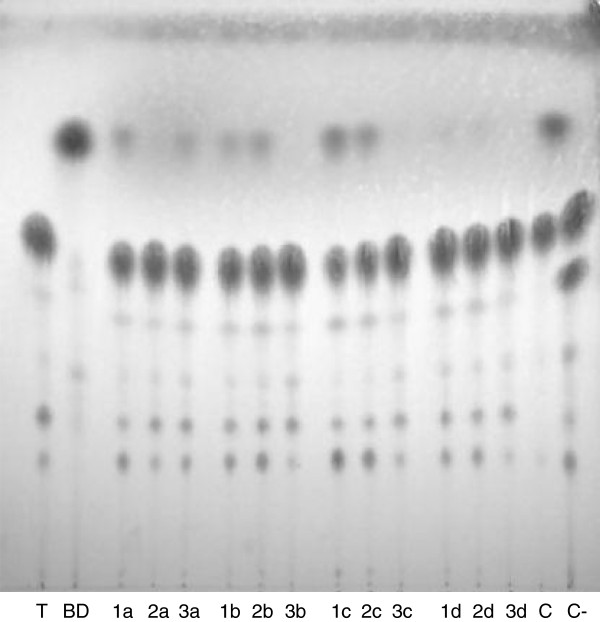
**TLC showing the products resulting from transesterification reactions.** T: triolein 65% (substrate); BD: industrial biodiesel (product control); C: transesterification reaction performed with Novozym 435, shown as a control; C-: negative control of a transesterification reaction run without alcohol. Enzymes immobilized on 1) EP100; 2) MP1000; 3) Celite®545. Reaction products of a) LipA; b) LipC; c) LipCmut; d) LipI.3. FAMEs production was clearly detected in lanes 1a, 3a, 1b, 2b, 1c, 2c and C, where spots at the same height of industrial biodiesel appeared.

As a possible biotechnological application, the four immobilized lipases were assayed in preliminary transesterification reactions for FAMEs production. The transesterification reaction conditions used were an adaptation of a previous protocol to the immobilized enzymes [26]. Impure triolein, (Sigma) containing also di- and monolein, was used as a substrate to simulate a natural vegetable oil, generally constituted by a mixture of glyceride forms. The reaction mixture contained the oil substrate, immobilized enzyme, and a defined amount of water and methanol, as described in materials and methods. The resulting products were analyzed through TLC (Figure 3), used as a fast and simple qualitative system for confirmation of FAMEs synthesis. A control sample showing the substrate impurity is visible in the first lane of Figure 3, where several spots appear, the most abundant corresponding to triolein. An industrially synthesized biodiesel sample, loaded in the second lane, was used as a control for FAMEs production. FAMEs appearance in course of the reaction with lipases LipA, LipC, LipCmut and LipI.3 can be identified in Figure 3 as spots migrating at the same position as the industrially synthesized biodiesel (lane 2). Although moderate, production of FAMEs by LipA, LipC and LipCmut preparations was detectable. Unfortunately, no FAMEs spots were revealed in the case of LipI.3-mediated transesterifications. Independently of the behavior of LipI.3, the highest yield in FAMEs production could be detected in reactions carried out with LipA-EP100, LipA-Celite®545 and with LipC and LipCmut immobilized on the two Accurel matrices, where discernible biodiesel spots appeared. Once demonstrated that new, non-commercial lipases can be efficiently immobilized on low-cost supports following a simple and fast protocol, and having shown that these lipases can perform transesterification reactions, further experiments are being conducted to increase the immobilized activity and to assess the re-usability of such preparations [10].

## Discussion

Lipases LipA, LipC, LipCmut and LipI.3 were efficiently immobilized on all assayed supports with no need for time-consuming and expensive purification protocols. Use of the selected strains as hosts for multicopy plasmid-encoded lipases allowed high enzyme production and permitted to skip the purification steps for each enzyme [27], thus reducing time and costs of the entire enzyme isolation process. Moreover, supernatants from PABST7.1 cultures generally produce a very low amount of total protein (ca. 0.033 mg mL^-1^), where each cloned lipase is the most abundant enzyme, as they are produced in higher copy number than any other secreted, chromosome-born protein. Therefore, the corresponding culture supernatants obtained can be considered as semi-purified enzyme preparations [10]. Cost and time savings in lipase isolation would be a great advantage in future scaling up of enzyme production for immobilization or biotechnological application. Although several attempts were performed to obtain higher expression yields of Lip I.3, low expression rates were obtained, indicating that new strategies should be developed for further lipase production improvement. Nevertheless, the amount of enzyme obtained by inclusion body refolding was sufficient for the immobilization assays performed.

In contrast to other immobilization procedures using sophisticated protocols and expensive supports [7,9], economic matrices were tested here to make the process industrially feasible. All four lipases tested were efficiently adsorbed on the Accurel matrices used, where a fast decrease of residual activity could be observed during the first 5 h incubation. For these supports, lipolytic activity of the batch supernatant was lost after 24 h incubation, indicating a complete immobilization. Immobilization on Celite®545 provided the best results for LipA. However, although the protocol for immobilization on Celite®545 is extremely fast, cheap and easy [9], the use of cold acetone in high proportions for enzyme precipitation could become a disadvantage during an eventual scaling up of the immobilization process. Therefore, we suggest polypropylene supports like Accurel MP1000 as the more appropriate carriers for lipases LipA, LipC, LipCmut and LipI.3, considering their possible role in biocatalysis.

It has been reported that adsorption occurs via hydrophobic interactions and is generally more effective for lipases than for other proteins [28]. Considering the high hydrophobicity shown by lipases we can assume that the highest proportion of proteins adsorbed on the supports assayed here were the lipases tested, with only minor adsorption of other proteins derived from supernatants or urea-dissolved cell extracts. It has also been reported that the success of hydrophobic materials in lipase adsorption depends on the structural features of “true” lipases, that is, those bearing a structural domain called “lid” [29]. The “lid” contributes to provide an open and a closed configuration to the enzyme. In the closed conformation, the “lid” exposes the hydrophobic residues towards the catalytic site, thus preventing substrate binding and activity. But in the presence of a hydrophobic interface, the “lid” changes its conformation and exposes the catalytic site to the substrate (triglyceride), in a phenomenon known as “interfacial activation” [29]. LipA, LipC and LipCmut possess a “lid” that covers the active site and is related to the affinity of lipases towards hydrophobic matrices [30]. Although LipA bears a “lid”, it does not show interfacial activation [21] a fact that could justify its different immobilization behavior with respect to LipC and LipCmut, which display interfacial activation and resulted here better immobilized on hydrophobic Accurel supports. Interestingly, LipI.3, belonging to a different lipase family, does not have a canonical “lid” but displayed the highest activity when immobilized on the hydrophobic support Accurel MP1000, probably due to a major exposure of its catalytic site. In the case of LipC and LipCmut, it is likely that during adsorption on polypropylene matrices, these lipases change their conformation in the presence of the hydrophobic support surface, being thus adsorbed in the open configuration and therefore being more active than when immobilized on Celite®545. To understand why immobilization of LipA on Accurel EP100 produced the lowest activity recovery, another important factor should be considered: the orientation of the adsorbed enzyme. Indeed, due to the hydrophobic nature of the support, it is possible that some lipase molecules interact with the support through the very hydrophobic surface surrounding the active site. This might hinder the substrate reaching of the active site [12], producing low activity values. It has also been shown that enzyme loading is directly correlated with the activity of the immobilized biocatalyst [31]. At this respect, it was reported that when *Thermomyces lanuginosus* lipase was immobilized on EP100 at low loading, part of the enzyme molecules were completely inactivated: with a loading of 6.6 mg of protein g^-1^ support, only 70% of the active sites were titrable compared to the 93% titrable active sites obtained at a loading of 55 mg g^-1^ support [15]. Moreover, immobilization might also lead to the inactivation of some lipase molecules by the distortion of the tertiary structure of the enzyme caused by the enzyme–support interaction. This would lead to structural rearrangements that could cause a decrease in lipase activity. All these effects may affect to a different extent each immobilized enzyme, thus making necessary to assay each individual lipase for immobilization efficiency on the desired support prior to application and scaling up for industrial purposes.

Immobilized lipases were evaluated for FAMEs production using triolein as a model substrate. LipA, LipC and LipCmut produced a visible FAMEs spot when analyzed by TLC, indicating that they are candidates for further study and process improvement. Enzyme or reaction engineering could contribute to increase the FAMEs yield obtained here. Lack of FAMEs formation with Lip I.3 could be explained in terms of substrate specificity, knowing that triolein is not a good substrate for this enzyme when used in hydrolysis reactions [23]. Alternatively, lack of transesterification activity could be due to a negative effect of immobilization on Lip I.3 when used in synthesis reactions, to a low enzyme concentration, or to inhibition by methanol, reasons that deserve further investigation.

The results obtained demonstrate that non-commercial lipases LipA, LipC, LipCmut and LipI.3 can efficiently be immobilized on all assayed supports, being polypropylene matrices (Accurel EP100, for LipA, and Accurel MP1000, for LipC and its variant) the best supports to be employed, especially in transesterification reactions, since they do not involve elaborate protocols, they are easy to recover and sufficiently cheap to be used in an eventual scaling up of the process. Immobilized LipA, LipC and LipCmut were capable to produce FAMEs from triolein under the conditions used here; therefore, they deserve further investigation.

## Conclusions

From the results obtained it can be concluded that it is possible to expand the existing enzyme toolbox by testing new, non-commercial enzymes produced by easy and fast procedures, which can further be immobilized by simple adsorption on economic supports. These methodologies can contribute to introduce new enzymatic tools for biotechnological applications such as biodiesel production, as they do not involve any time or solvent consumption, they are easy to conduct and sufficiently economic to be used in an eventual scaling up.

## Methods

### Materials and reagents

All chemicals and solvents were obtained from Sigma Aldrich and Serviquimia (Spain) and, except when stated, had quality for analysis. Accurel EP100 (particle size 200–500 μm) was from Azko Nobel (Obernburg, Germany; kindly provided by Dr. F. Valero). Accurel MP1000 (particle size under 1500 μm) was purchased from Membrana GmbH (Wuppertal, Germany). Celite®545 (diatomaceous earth, 30–80 mesh) was obtained from Sigma-Aldrich.

### Bacterial strains and enzyme production

*Pseudomonas* strains and the plasmids used in this work are listed in Table 2. *Escherichia coli* 5 K, used as recipient strain for recombinant plasmids, was grown in Luria–Bertani medium (LB, Panreac, Spain) at 37°C and supplemented with 50 μg chloramphenicol mL^-1^ when necessary. *Pseudomonas* strains were routinely grown in LB medium (supplemented with 400 μg mL^-1^ chloramphenicol and 50 μg mL^-1^ tetracycline, when applied) at 30°C on a reciprocal rotary shaker (180 rev min^-1^). *Pseudomonas* PABST7.1 is a PAO1 mutant deficient for *lip*A and *lip*H genes, where LipC is inactive due to the lack of the specific foldase LipH [32].

**Table 2:** Strains used

	**Strain**	**Features**	**Reference**
	*Pseudomonas* sp. 42A2	Wild type	[33]
	*Pseudomonas* sp. CR-611	Wild type	[24]
	*P. aeruginosa* PABST7.1	Δ*lip*A Δ*lip*H miniD-180 (*tet*A *tet*R *lac*Iq P*lac*UV5-T7 gene1)	[32] Kindly provided by Dr. Rosenau.
**LipA**	*P. aeruginosa* PABST7.1 lipAHpBB	Contains *lip*A and *lip*H 42A2 gene	[21]
**LipC**	*P. aeruginosa* PABST7.1 lipCHpBB	Contains *lip*C and *lip*H 42A2 gene	[22]
**LipCmu**t	*P. aeruginosa* PABST7.1 lipCHpBB (variant D2_H8)	Contains mutated *lip*C and wild type *lip*H 42A2 gene	[22]
**LipI.3**	E. coli 5 K (pGEM-T Lip I.3)	Contains *lip*I.3 CR-611gene	[23]

*Pseudomonas sp.* 42A2 lipases, LipA and LipC [21,33], and the thermostable variant LipCmut [22] were previously cloned with their specific foldase (LipH) in pBBR1MCS vector [34] and transformed into homologous *Pseudomonas* PABST7*.*1. All of them are naturally secreted lipases [21] and the enzyme recovery was easily carried out by centrifugation of the culture broth (20 min at 8000 rpm).

LipI.3, isolated from *Pseudomonas* sp. CR-611 [35], was previously cloned in pGEMT and transformed into *E. coli* 5 k [23] where it is expressed as inclusion bodies in an inactive form. Inclusion bodies were obtained by French press cell-disruption and solubilised according to Kojima’s method [36]. Using a refolding protocol with urea 8 M, active LipI.3 was obtained in phosphate buffer 50 mM pH 7, after urea removal by dialysis [23].

### Enzyme immobilization

Direct culture supernatants were used for LipA, LipC and LipCmut immobilization, whereas LipI.3 was immobilized from a solubilised inclusion body solution, thus skipping the high costs and time-consuming steps of protein purification.

#### Adsorption on EP100 and MP1000

Supports were pretreated following an adaptation from Guillen and coworkers [37] where the polypropylene matrices were activated with ethanol (3 mL per gram of MP1000 or EP100), followed by alcohol removal by vacuum filtration. Immobilization was carried out by mixing the pretreated supports with 5 mL cell extract or supernatant samples, following a ratio of 1 mg protein per g of EP100 and 2 mg protein per g of MP1000. Orbital shaking of the mixture was carried out for 24 h at each enzyme’s optimum temperature, being 4°C for LipC and LipCmut, and 30°C for LipA and LipI.3. Control samples were run without any support under the same conditions. Supernatant samples from immobilization mixtures were taken at different times and their residual lipase activity was assayed as described [38]. The enzyme-loaded supports, containing the immobilized lipases, were recovered by vacuum filtration, dried in a speed vacuum and stored at 4°C after lipase activity determination.

#### Adsorption on celite®545

Immobilization on Celite®545 did not require any pretreatment and was performed by mixing 5 mL of cell extracts or supernatants with the support, using a ratio of 0.5 mg protein per g Celite®545. The mixture was incubated for 30 min at 4°C and precipitation of the enzyme onto the support was carried out with 5 mL cold acetone. Immobilized enzymes were then collected by vacuum filtration and dried with a speed vacuum concentrator prior to enzyme activity determination and storage at 4°C [9].

### Activity assays

Activity of free enzyme in cell extract and supernatant fractions, or in supernatants from the immobilization mixture was analyzed by measuring the release of *para*-nitrophenol (*p*NP) from *p*NP-derivative fatty acid substrates, as a result of enzymatic hydrolysis, measured at λ = 405 nm as previously described [21,38]. One unit of activity was defined as the amount of enzyme that released 1 mol of *p*NP per minute under the assay conditions used. Lipase activity was routinely assayed at the optimum temperature for each enzyme (30°C for LipA and LipI.3; 4°C for LipC and LipCmut), using the standard conditions: 20 mM Tris HCl buffer at optimum pH of each enzyme (pH 8 for LipA, LipC and LipCmut; pH 5.5 for LipI.3), and 0.6% Triton X-100®. CaCl_2_, at a final concentration of 20 mM, was added to the reaction mixture for LipI.3 activity assays [23].

Activity of immobilized lipases was measured using 2 mg support carrying the immobilized enzyme adsorbed on EP100 and MP1000, and 10 mg enzyme-loaded support for Celite®545. The reaction mixture was incubated at each enzyme’s optimum temperature and was stopped by 2 min centrifugation at 14,000 rpm. Activity determination was carried out in 400 μL Tris 100 mM plus 100 μL *p*NP-buffer, as described above. One unit of immobilized lipase activity was defined as the amount of enzyme that released 1 mol of *p*NP per minute and per g of support, under the assay conditions used [21,38]. Protein concentration of free and immobilized samples was determined as previously described [39]. When required, dilution of lipase solutions was done using buffer Tris 20 mM pH 8 for LipA, LipC and LipCmut, and pH 7 for LipI.3 [21,23].

### Transesterification assays

FAMEs synthesis reactions were carried out in 2 ml glass vials for 24 h with maximum agitation in a horizontal vortex. LipC and LipCmut reactions were carried out at room temperature, according to the cold-adapted behavior of these enzymes [21,22]. A temperature of 30°C was used for LipA and LipI.3 [21,23]. The reaction mixtures contained 1 g triolein of 65% purity to simulate the composition of mixed vegetable oils. Methanol 15% w/w of oil was used as the second substrate, and was added in two steps (7.5% each) at 0 h and 7 h, to prevent possible enzyme inhibition. Water (10% w/w of oil) was added to the reaction mixture to increase enzyme efficiency [26] and, for LipI.3, 20 mM CaCl_2_ was also added [23]. In order to compare the effectiveness of immobilized lipases in transesterification assays, all enzymatic preparations were loaded of 2U lipase, calculated from their hydrolytic activity when immobilized. As positive control, the same reaction conditions were applied to a commercial immobilized lipase (Novozym 435, Novozymes DK), whereas in negative controls, the same reaction was performed without methanol for detection of possible hydrolysis processes. After 24 h reaction, the products were evaporated in a speed vacuum concentrator at 60°C for 1½ h to remove excess methanol and the upper layer, obtained by centrifugation during the same evaporation process, was prepared for TLC analysis.

### Thin layer chromatography

AluGram UV254 silica gel plates (Mecherey Nagel) were used as stationary phase for TLC analysis, and a mixture of hexane-diethyl ether-acetic acid (80:20:1.5 v/v/v) was used as the mobile phase. Commercial triolein (Sigma) and a sample of industrially synthesized biodiesel (BE- Biodiesel, Spain) were used as standards for glycerides and FAMEs, respectively. Enzymatic reaction products and standards were diluted 1:50 in hexane, and 8 μL samples were then applied to a 20 × 20 cm plate. 15% phosphomolibdic acid in ethanol was used as color developer through a fine spray. Spot visualization was completed by mildly heating the plate with an air dryer.

## Competing interests

No interest or financial relationships that could influence the author’s objectivity exist regarding the information provided in it. Neither patent or stock ownership, membership of a company board of directors, membership of an advisory board or committee for a company, and consultancy for or receipt of speaker's fees from a company apply to the manuscript authors who are a Post-doctoral fellow, a PhD student, and two public University professors. Therefore, the authors declare that they have no competing interests.

## Authors’ contribution

SC participated in the design of the study, carried out most of the experiments, organized and interpreted the data, and drafted the manuscript. BI made some of the immobilization experiments, performed the TLC assays and contributed to the manuscript draft. FIJ P contributed to critical discussion, and revised the manuscript. PD coordinated the project and the design of the study, critically interpreted the data and corrected the manuscript. All authors read and approved the manuscript.
